# Medical Treatment of Gastric Ulcer

**Published:** 1917-07

**Authors:** Geo. P. Hamner

**Affiliations:** Lynchburg, Va.


### MEDICAL TREATMENT OF GASTRIC ULCER.

By Geo. P. Hamner, M.D., Lynchburg, Va.

Following the time-honored method of medical treatment for this condition, there arose a surgical age for gastric ulcer. This began when it was discovored that the abdominal cavity could be invaded with impunity under proper asepsis, and for a time almost every diagnosed ulcer of the stomach came to the surgeon when the consent of the patient could be gained. The brilliant results hoped for were by no means always obtained from operation, and the pendulum has again swung back to at least a faithful trial by the internists, for healing before surgery is resorted to. This is as it should be, for it is a well known fact that gastric ulcers are frequently multiple, and sometimes inoperable, and, again, there is a tendency to recurrence in other parts of the gastric mucosa even after excision.

The expectant treatment for the healing of ulcer is first, and most important of all, rest in bed, next to which comes diet, and, lastly, medication.

The rest cure should last from ten days to four weeks, according to the severity of the symptoms. The diet should be divided into about four periods. For the first ten days it should consist if not exclusively of liquids,—milk, buttermilk, nutritious soups, bouillon and meat extracts. Cases in which these are not well borne are exceptional. Broths made of wild game are usually grateful to the palate and well toleraed by the stomach.

In mild cases, when nausea and pain are of a minimum type, semi-solids may be begun during this period. The feeding should, of course, be fractional, the intervals being from two to four hours. During the second period, of about seven days, the diet, in addition to liquids, may consist of two or three cakes (old-fashioned sweet cookies), soft eggs, rice, breasts of tender chicken, pigeon or quail and sweet-breads.

The third period: Tea, coffee, or cocoa with milk and sugar, English biscuits, scraped, rare steak, mashed potatoes, scraped ham. All solids should be finely divided, and the patient cautioned to masticate thoroughly. "Eat slowly and chew thoroughly" is a good slogan.

The fourth period: A bland diet, based on a test breakfast analysis, acid free and with a minimum amount of sweets, should be prescribed, to be continued until a complete cure is affected.

Medication: When not too exhausting to the patient, and there is little or no bleeding produced, it has become my practice to begin lavage at once, cautiously, with a soft rubber tube, using alkaline solutions, and frequently running in at the last from one-half to one pint of 1-6,000 or 1-8,000 silver nitrate solution, which is left in the stomach. Also a solution of bismuth subnitrate about the consistency of buttermilk to fill up the ulcer and leave a smooth coating over the inflamed area around it is advisable.

If one is cautious in passing the tube and the operation is not extremely distressing to the patient, the danger of harm is almost nil.

I have seen persistent vomiting yield to this method time and time again when every other procedure had failed, and the result is frequently immediate and most gratifying.

As in all other diseases in which little result is obtained from medication, the list of drugs that have been vaunted for the treatment of gastric ulcer is exhausive. Personally, I have little faith in any of them. Nitrate of silver with hyoscyamus in pills, or bismuth subnitrate, might be tried, but the healing of a gastric ulcer is a reconstructive process and must be accomplished by the tissues concerned.

Keep the wounded muscosa clean, free from mechanical irritation, the gastric secretions as nearly alkaline as possible, give the ulcer a fair chance to heal, with the patient under close observation, and if the case is not progressing satisfactorily, do not hesitate to resort to surgical methods promptly, or you may suffer the humiliation of a perforation and lessen greatly the chance of curing your patient.—The Virginia Medical Semi-Monthly.

